# Expressions of mRNA and encoded proteins of mitochondrial uncoupling protein genes (UCP1, UCP2, and UCP3) in epicardial and mediastinal adipose tissue and associations with coronary artery disease

**DOI:** 10.20945/2359-3997000000582

**Published:** 2023-01-17

**Authors:** Claudia Huesca-Gómez, Yazmín Estela Torres-Paz, Giovanny Fuentevilla-Álvarez, Nadia Janet González-Moyotl, Edgar Samuel Ramírez-Marroquín, Xicótencatl Vásquez-Jiménez, Víctor Sainz-Escarrega, María Elena Soto, Reyna Samano, Ricardo Gamboa

**Affiliations:** 1 Instituto Nacional de Cardiología “Ignacio Chávez” Departamento de Fisiología, Ciudad de México Ciudad de México México Instituto Nacional de Cardiología “Ignacio Chávez”, Departamento de Fisiología, Ciudad de México, México; 2 Instituto Nacional de Cardiología “Ignacio Chávez” Departamento de Cirugía Cardiotorácica Ciudad de México México Instituto Nacional de Cardiología “Ignacio Chávez”, Departamento de Cirugía Cardiotorácica, Ciudad de México, México; 3 Instituto Nacional de Cardiología “Ignacio Chávez” Departamento de Inmunología Ciudad de México México Instituto Nacional de Cardiología “Ignacio Chávez”, Departamento de Inmunología, Ciudad de México, México; 4 Instituto Nacional de Perinatología Coordinación de Nutrición y Bioprogramación Ciudad de México México Instituto Nacional de Perinatología, Coordinación de Nutrición y Bioprogramación, Ciudad de México, México

**Keywords:** Mitochondrial uncoupling proteins, mediastinal adipose tissue, epicardial adipose tissue, cardiovascular disease

## Abstract

**Objective:**

To evaluate the expression of UCP1, UCP2, and UCP3 mRNA and encoded proteins in epicardial and mediastinal adipose tissues in patients with coronary artery disease (CAD).

**Subjects and methods:**

We studied 60 patients with CAD and 106 patients undergoing valve replacement surgery (controls). Expression levels of UCP1, UCP2, and UCP3 mRNA and encoded proteins were measured by quantitative real-time PCR and Western blot analysis, respectively.

**Results:**

We found increased UCP1, UCP2, and UCP3 mRNA levels in the epicardial adipose tissue in the CAD versus the control group, and higher UCP1 and UCP3 mRNA expression in the epicardial compared with the mediastinal tissue in the CAD group. There was also increased expression of UCP1 protein in the epicardial tissue and UCP2 protein in the mediastinum tissue in patients with CAD. Finally, UCP1 expression was associated with levels of fasting plasma glucose, and UCP3 expression was associated with levels of high-density lipoprotein cholesterol and low-density cholesterol in the epicardial tissue.

**Conclusions:**

Our study supports the hypothesis that higher mRNA expression by UCP genes in the epicardial adipose tissue could be a protective mechanism against the production of reactive oxygen species and may guard the myocardium against damage. Thus, UCP levels are essential to maintain the adaptive phase of cardiac injury in the presence of metabolic disorders.

## INTRODUCTION

Fat accumulation around the heart has been established as a risk factor for various cardiovascular diseases. In the cardiac region, fat accumulation occurs inside and outside the pericardium in fatty deposits in the epicardial and paracardial areas, the latter corresponding, essentially, to the mediastinal adipose tissue (MAT). The epicardial adipose tissue (EAT) has been the target of various investigations due to its relationship with the coronary arteries and myocardium and potential influence on the development of coronary artery disease (CAD). The EAT, which comprises visceral fat below the visceral pericardium and in close contact with the coronary arteries ( 1 ), contains many fatty acids that are free and capable of secreting proinflammatory and proatherogenic cytokines and antiatherogenic adipocytokines ( 1 ). Although the EAT has a complex and incompletely understood functional role, it probably has multiple functions, including mechanical, metabolic, thermogenic, and endocrine/paracrine roles ( 2 ). Compared with subcutaneous and visceral fat deposits, the EAT has been reported to have a higher rate of release and absorption of free fatty acids (FFAs) ( 3 ). Since the myocardial metabolism is highly dependent on FFA oxidation, the EAT provides the myocardial energy needs, especially during periods of high demand.

Uncoupling proteins (UCPs) 1, 2, and 3 are members of an anion-carrier protein family with structural similarities, located in the mitochondrial inner membrane and carrying tissue-dependent genetic expression in mammals ( 4 ). The primary function of the UCPs is to uncouple the mitochondrial oxidative phosphorylation, promoting leakage of protons across the inner mitochondrial membrane without passing through the charge pathway to synthesize ATP (a process involved in producing heat). As a result of this uncoupling mechanism, stored triglycerides are mobilized and play an essential role in fat metabolism ( 4 , 5 ). Some publications have shown that UCP expression by the related genes is higher in the EAT than in other fat deposits in the body, such as the abdomen, thighs, and subcutaneous tissue ( 6 ). Also, some studies have shown that polymorphisms of the *UCP* genes may contribute to metabolic disorders with significant effects on energy metabolism ( 7 ), directly and indirectly affecting obesity and type 2 diabetes phenotypes ( 8 – 10 ).

Based on these considerations, the main aim of our study was to evaluate the expressions of *UCP1, UCP2* , and *UCP3* mRNA and encoded proteins in EAT and MAT and their associations with CAD.

## SUBJECTS AND METHODS

### Population

**Figure 1:**
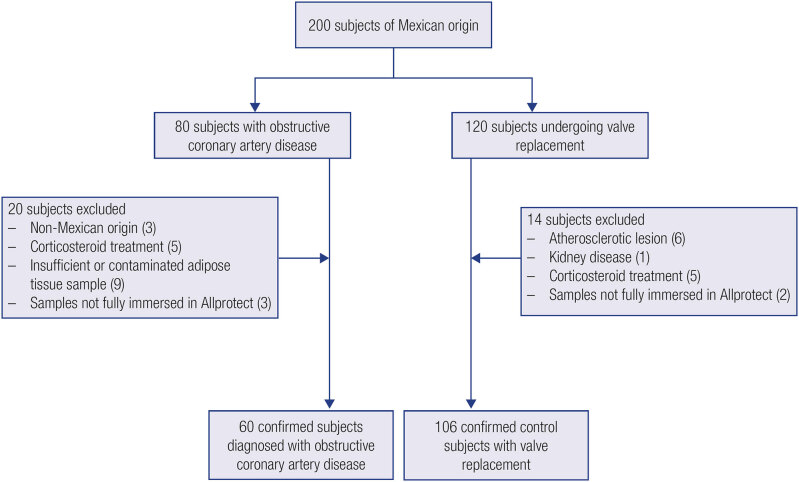
Flowchart of study sample selection.

This case-control study included 166 adult patients of both sexes and older than 18 years who agreed to participate in the research. Of these, 60 had undergone elective primary coronary artery bypass surgery for treatment of angiographically proven obstructive CAD ( 11 ), defined as a disease causing stress- or exercise-related symptoms of angina due to a narrowing of ≥ 50% in the left common trunk or ≥ 70% in one or more of the major arteries. The remaining 106 patients (control subjects) had undergone valve replacement unrelated to atherosclerosis lesions and had normal coronary angiography in the preoperative period. Patients with liver or kidney disease, cancer, untreated thyroid dysfunction, or infectious conditions and those on corticosteroid treatment were excluded from the study in both groups. All participants answered standardized and validated questionnaires collecting information on family and medical history, physical activity, and alcohol and tobacco consumption. The exclusion criteria were non-Mexican origin, corticosteroid treatment, and contaminated or insufficient samples ( Figure 1 ). Diabetes mellitus was defined as glucose values ≥ 126 mg/dL for at least 3 months. Dyslipidemia was defined as abnormal lipid levels; alcoholism was defined according to the Michigan Alcoholism Screening Test (MAST) ( 12 ) and AUDIT trail ( 13 ). Smoking was described as smoking of at least one cigarette per day in the previous 6 months.

### Sample size

The sample size was calculated considering that, at the National Institute of Cardiology “Ignacio Chávez” in 2018, 120 coronary revascularization surgeries were performed, and a total of 155 patients underwent valve replacement surgery. The sample size was calculated according to differences in means between groups, considering an incidence of the *UCP* gene of approximately 0.08 in the cases and 0.02 in the controls with a Δ = 0.06, statistical power of 95%, and p < 0.05. According to the following formula, our “n” value was 59:


$$\begin{array}{c} poqo\left[Z\propto +Z\beta \sqrt{\frac{piqi}{poqo}}\right]2\\ (\bar{pi-po})2 \end{array}$$

po = Probability that *UCP* expression occurs in cases

q0 = Probability that *UCP* expression does not occur in cases

pi = Probability that *UCP* expression occurs in controls

qi = Probability that *UCP* expression does not occur in controls

1.96 = value < 0.05

1.28 = power (0.84)

### Ethical aspects

Signed informed consent was obtained from each participant after a full explanation of the purpose and nature of all procedures used in the research study, as recommended in the Declaration of Helsinki, modified in the Tokyo Congress, Japan ( 14 ). The research was approved by the Ethical, Biosecurity, and Investigation Committees of the National Institute of Cardiology (registration number 10-690).

### Biochemical analysis

Blood samples were collected by venipuncture after 12-hour fasting. Levels of total cholesterol, triglycerides, high-density lipoprotein cholesterol (HDL-C), low-density lipoprotein cholesterol (LDL-C), and glucose were measured using the Lowry 2000 enzymatic photometric system DiaSys (DiaSys Diagnostic Systems, Holzheim, Germany).

### Epicardial and subcutaneous adipose tissue

Biopsies from adipose tissues were obtained during revascularization or valvuloplasty surgery, depending on the group. Samples of EAT (0.5 to 1.0 g) were obtained from the region proximal to the left anterior descending coronary artery due to the presence of lesions in different segments, and samples of MAT were obtained from the pectoral region. Each EAT and MAT sample was sectioned into two portions, placed in tubes with Allprotect Tissue Reagent (QIAGEN, Hilden, Germany), and frozen at -70 °C until RNA and protein extraction.

### Quantification of mRNA by real-time PCR

According to the manufacturer's protocol, RNA and protein were isolated using TriPure Isolation Reagent (Roche Molecular Biochemicals, UK). Reverse transcription reaction (RT-qPCR) was performed using 1 µg of total RNA for cDNA synthesis according to the High-Capacity cDNA Reverse Transcription Kit (Applied Biosystems, Foster, CA, USA). cDNA was stored at -80 °C. The quantification of mRNA (qPCR) was accomplished using the Bio-Rad CFX96 Real-Time System (Bio-Rad, Hercules, CA, USA). Levels of *UCPs* and *HPRT* (Hs99999909_m1) (reference gene) expression were measured using a commercially available kit (TaqMan Gene Expression Assay, Applied Biosystems). The specificity and the optimal primer and probe concentrations were tested. Amplifications were performed starting with a 10-minute template denaturation step at 95°C, followed by 40 cycles at 95°C for 15 seconds and 60 °C for 1 minute. No reverse transcriptase (NRT) controls were prepared for each sample. First-strand cDNA samples were stored at -20°C. The primer sets used targeted the following human genes: *UCP1* (Hs01084774_m1), *UCP2* (Hs01075227_m1), and *UCP3* (Hs01106052_m1). Gene expression levels were quantified in duplicate, and the quantitative cycle (Cq) values were determined and normalized using the reference gene expression ( *HPRT* ). Reaction efficiencies were determined from the standard curves. Standard curves for each target were prepared from pooled samples of first-strand cDNA from CAD patients and valve patients (controls). Four randomly selected cDNA samples from each group were pooled, and eight serial dilutions were made. All data were expressed relative to each control value. Relative quantification was carried out by means of the formula 2^−ΔΔCt^ ( 15 ).

### Western blot analysis

Proteins were analyzed by Western blot analysis as previously described ( 16 ). Blots were incubated with antibodies against human UCP1 (polyclonal antibody Abcam ab10983, dilution 1:500), human UCP2 (polyclonal antibody Abcam ab67241, dilution 1:500), and human UCP3 (polyclonal antibody Abcam 10985, dilution 1:1000). Equal loading of protein in each lane was verified by staining filters with Ponceau and incubating blots with monoclonal antibodies against human β-actin (Abcam ab8226).

The Quantity One software (Bio-Rad) was used to quantify the membrane bands via densitometry, which were detected using Clarity Western ECL Substrate (Bio-Rad). The expression levels were measured in duplicate and normalized and then compared against the concentration of a loading protein control. The results were expressed as arbitrary units of intensity.

### Statistical analysis

Data were analyzed using SPSS, v21 (SPSS Inc., Chicago, USA). The sample size calculation considered a confidence interval of 95% and power of 84%. The results are presented as mean ± standard deviation (SD) for variables with parametric distribution. Variables without normal distribution are presented as median (minimum-maximum) values. The Kruskal-Wallis test was carried out to compare two or more independent samples. The Shapiro-Wilk test was used to assess normality. Comparisons between groups were performed using the unpaired Student's *t* test for continuous variables and the Mann-Whitney U test for discontinuous variables. For categorical variables, the chi-square test was used. Univariate analysis was performed using one-way analysis of variance (ANOVA) to evaluate outcomes in the control group and differences in clinical variables among patients. Subsequently, binary logistic regression analysis was carried out to explore the association between UCP expression and the main factors associated with CAD. A p value < 0.05 was considered statistically significant.

## RESULTS

### Characteristics of the study population

In the study population of 166 subjects, 60 patients were in the CAD group and 106 were in the valve replacement group (controls). Table 1 shows the biochemical and anthropometric characteristics of the participants. Patients with CAD, compared with controls, had lower serum levels of HDL-C (p = 0.039) and higher serum levels of triglycerides (p = 0.011) and glucose (p < 0.001), along with increased rates of diabetes (p = 0.004), smoking (p < 0.001), and use of statins (p < 0.001) and anti-hypertensive drugs (p = 0.012).

**Table 1:** Biochemical and anthropometric characteristics of the study population

Characteristics (n = 166)	Patients with CAD (n = 60)	Patients with valve disease (n = 106)	P values
Age (years)	59.87 ± 9.76	51.14 ± 15.5	**0.290**
Sex – men/women (%)	87.5/12.5	84.2/15.8	**0.570**
BMI (kg/m^2^)	26.6 ± 3.1	26.4 ± 4.3	**0.328**
TC (mg/dL)	156.2 ± 56.5	155.0 ±42.8	**0.124**
HDL-C (mg/dL)	35.3 ± 7.9	40.3 ± 13.3	**0.039**
LDL-C (mg/dL)	98.0 ± 46.5	90.2 ± 31.1	**0.120**
Triglycerides (mg/dL)	179.5 ± 88	131.2 ± 56.3	**0.011**
Glucose (mg/dL)	116.2 ± 37.8	99.8 ± 18.8	**0.001**
SBP (mmHg)	110 ± 12	113 ± 11	**0.791**
DBP (mmHg)	69 ± 7	70 ± 9	**0.675**
Dyslipidemias (%)	15.0	23.5	**0.187**
T2DM – plasma glucose ≥ 126 mg/dL (%)	32.2	13.2	**0.004**
Current smoking (%)	63.6	34.7	**0.000**
Current alcoholism (%)	20.0	19.8	**0.098**
Hypertension – BP ≥ 140 mmHg (%)	50.0	41.5	**0.290**
Use of statins (%)	52.3	8.4	**0. 001**
Use of antihypertensive drugs (%)	61.6	41.5	**0.012**
Use of hypoglycemic drugs (%)	8.3	2.8	**0.111**

Data are expressed as mean ± standard deviation values (when analyzed with the Student's *t* test) or percentages (when analyzed with the chi-square test).

CAD: coronary artery disease; BMI: body mass index; TC: total cholesterol; HDL-C: high-density lipoprotein cholesterol; LDL-C: low-density lipoprotein cholesterol; SBP: systolic blood pressure; DBP: diastolic blood pressure; T2DM: type 2 diabetes mellitus; BP: blood pressure.

Between men and women, no significant differences were observed regarding all parameters, except for levels of HDL-C (p = 0.035, data not shown).

### Expression of mRNA of *UCPs*

**Figure 2:**
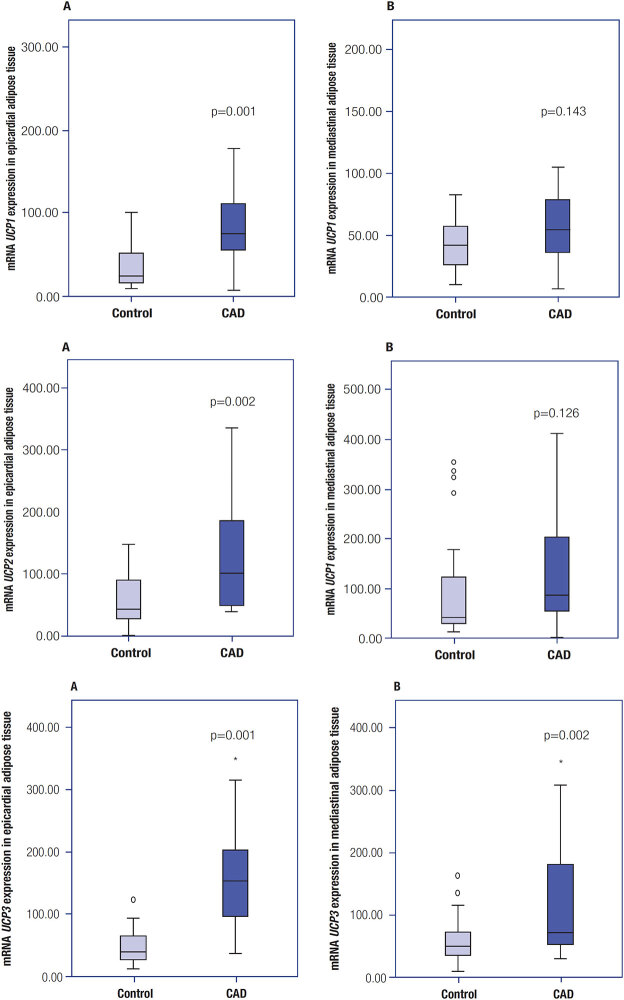
Levels of *UCP1, UCP2* , and *UCP3* mRNA expression in ( **A** ) epicardial adipose tissue and ( **B** ) mediastinal adipose tissue. The data were normalized against HPRT-1; expression are represented as median (minimum-maximum) values.

To determine if *UCP1, UCP2* , and *UCP3* mRNA and protein expression levels were a potential source of cardiovascular disease, we analyzed these levels in EAT and MAT obtained from the CAD and the control group. Figure 2 , displaying the levels of mRNA expression (in median [minimum-maximum] values), shows a higher expression of *UCP1* mRNA in EAT of patients with CAD versus controls (74.2 [6.6-179.1] versus 24.4 [9.9-100.9], respectively; p = 0.001). A similar pattern was observed for *UCP2* mRNA in EAP (102.6 [51.3-324.7] versus 45.8 [22.7-145.5]), respectively; p = 0.002) and *UCP3* mRNA in EAP (154.0 [35.4-316] versus 37.9 [11.1-91.2], respectively; p = 0.001]) and MAT (70.8 [29.1-307.6] versus 49.1 [10.1-110.2], respectively; p = 0.002).

**Figure 3:**
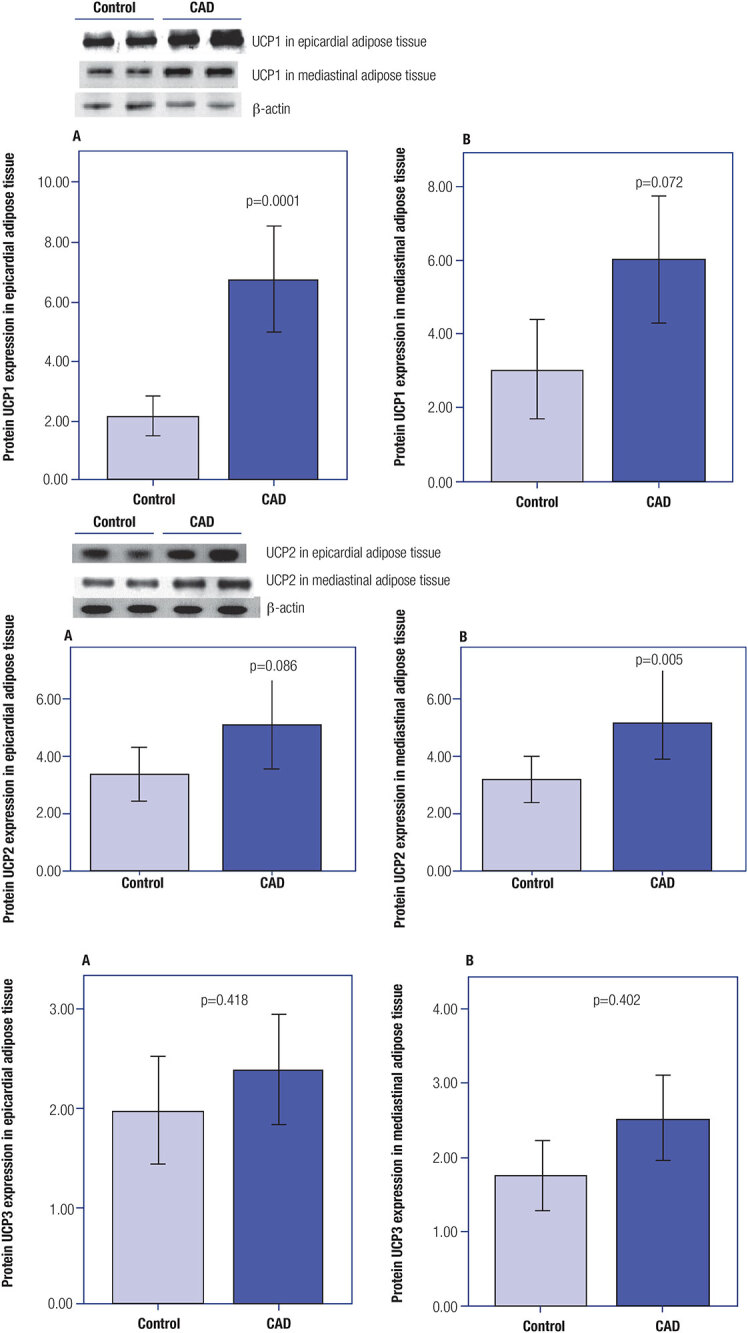
UCP1, UCP2, and UCP3 protein expression in the ( **A** ) epicardial adipose tissue and ( **B** ) mediastinal adipose tissue. The bands show the UCP1 protein expression in the epicardial adipose tissue and the UCP2 protein expression in the mediastinal adipose tissue. The β-actin bands were used as loading control.

Regarding protein expression, we also compared both groups and analyzed the expression levels in EAT and MAT separately ( Figure 3 ). We found a higher UCP1 protein expression in EAT in the CAD compared with the control group (6.8 ± 4.0 versus 2.2 ± 1.6, respectively; p = 0.0001) and a higher UCP2 protein expression in MAT in the CAD group compared with the control group (5.2 ± 3.8 versus 3.2 ± 2.0, respectively; p = 0.005).

To test for potential correlations between cardiovascular disease and adipose tissue region, we evaluate the expression of each *UCP* in EAT and MAT in both groups.

**Figure 4:**
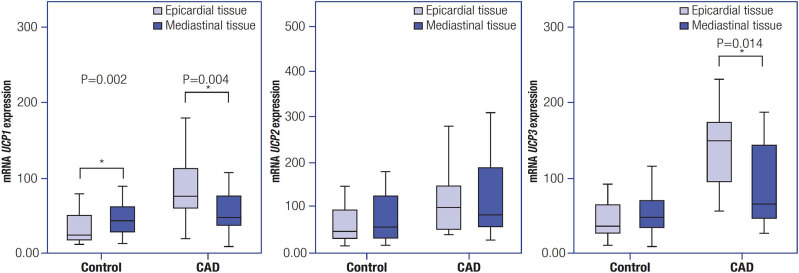
Levels of *UCP1, UCP2* , and *UCP3* mRNA expression in the epicardial and mediastinal adipose tissues in the CAD and control groups.

Figure 4 shows the levels of *UCP1, UCP2* , and *UCP3* mRNA expression in EAT and MAT in the CAD and control groups. In the CAD group, we observed a higher expression of both *UCP1* and *UCP3* in EAT compared with MAT (p = 0.004 for *UCP1* and p = 0.014 for *UCP3* ). In the control group, we observed the opposite, *i.e.* , overall increased expression in MAT compared with the EAT, but with a significant difference for mRNA of *UCP1* only (p = 0.002).

We also analyzed the correlation between *UCP1, UCP2* , and *UCP3* mRNA expression and laboratory parameters (serum levels of total cholesterol, triglycerides, LDL-C, and HDL-C) and clinical parameters comprising the main factors associated with CAD (hypertension, diabetes, and body mass index). We found significant associations with fasting plasma glucose levels for *UCP1* mRNA expression in EAT (p = 0.042, r = 0.8), with triglycerides in MAT (p = 0.004, r = 0.96), with HDL-C in *UCP3* in EAT (p = 0.007, r = 0.9), and with LDL-C in EAT (p = 0.025, r = 0.9) and MAT (p = 0.046, r = 0.8).

We found no differences in the expression level of the analyzed proteins after categorizing the patients by sex, age, and use of statins or anti-hypertensive drugs (data not shown).

## DISCUSSION

In the present study, we found significantly higher levels of fasting plasma glucose, LDL-C, and triglycerides and significantly lower levels of HDL-C in the CAD group compared with the control group, confirming interrelated damaging processes during cardiometabolic injury ( 17 ). We also observed increased expression levels of *UCP1, UCP2,* and *UCP3* mRNA and encoded proteins in the CAD compared with the control group. Among patients with CAD, this increased expression was more significant in the EAT compared with the MAT.

Different mechanisms – cytotoxicity, endocrine inflammation, oxidative stress, endoplasmic reticulum stress, and mitochondrial dysfunction – have been described to explain metabolic abnormalities ( 18 – 20 ), although, in recent decades, lipotoxicity has become the most widely accepted mechanism of all ( 18 , 21 ). The EAT plays an essential role in supplying fatty acids to the myocardium. Under normal conditions, the EAT regulates the homeostasis of fatty acids to prevent lipotoxicity and secretes anti-inflammatory and antiatherogenic adipokines ( 22 – 25 ). Dysfunction in EAT leads to adipokine changes that cause the release of fatty acids and proinflammatory cytokines in pathological situations, leading to CAD ( 8 , 26 , 27 ). The resulting inflammation process may induce the conversion of brown adipose tissue to white adipose tissue, which also secretes various proinflammatory cytokines, aggravating local and systemic inflammation and resulting in metabolic deterioration and damage.

Cardiac expression of *UCP2* is increased in patients with heart failure. However, the underlying causes for and possible consequences of this change during the transition from hypertrophy to heart failure are still unclear ( 28 ). A study with isolated ventricular cardiomyocytes in an adult rat model of hypertension has suggested that downregulated *UCP2* is associated with greater glucose absorption, indicating a metabolic change. However, during progression and decrease in ejection fraction, ventricular hypertrophy was accompanied by increased expression of *UCP2* mRNA ( 29 , 30 ). The authors of the study showed a biphasic expression of the UCP2 protein, with an initial decrease in the expression of this protein during the onset of hypertrophy followed by a significant increase afterward.

Previous studies have shown that reactive oxygen species (ROS) are the most important factor regulating the activity and mRNA expression of *UCP2* and *UCP3* ( 31 , 32 ). Production of oxygen free radicals in cardiomyocytes increases rapidly due to damage to the antioxidant systems during an event of myocardial ischemia and due to their increase in the bloodstream and supply of large amounts of oxygen to the cells of the ischemic area during reperfusion ( 33 , 34 ). Therefore, an elevated level of these proteins can be attributed mainly to high amounts of ROS during myocardial damage ( 34 ). On the other hand, *in vivo* and *in vitro* studies have shown that expression of UCPs increases during oxidative stress as a feedback response aimed at reducing ROS production ( 35 ). Consequently, increased UCP expression may act as a protective mechanism to decrease ROS production. These data could explain the increased expression of *UCP* mRNA and encoded proteins in our patients with cardiovascular damage, in which a condition of oxidative stress and release of ROS could lead to increased expression of these proteins. Also, other studies have detected a sequence before the promoter of *UCP2* and *UCP3* genes that contains a binding site for ROS-sensitive factors. Therefore, under oxidative stress, expression of *UCP* genes can also be increased at the transcriptional level ( 31 , 36 ).

The increased mRNA expression of *UCP* genes could be an adaptive protective response against cardiomyocyte damage. Safari and cols. ( 37 ) have reported that during various periods of ischemia and reperfusion in rat hearts, cardiac mitochondria showed more hydrogen leak, less membrane potential, less ATP content, more oxygen consumption, and considerably lowered ROS production. Also, levels of *UCP2* and *UCP3* mRNA and their corresponding proteins would produce ROS under anoxia and reperfusion ( 6 , 29 , 30 ). Browning of white adipose tissue has also been suggested to be an adaptive mechanism to alleviate redox pressure ( 38 ), with an increased expression of oxidative stress-related proteins in the EAT relative to subcutaneous adipose tissue in patients undergoing cardiac surgeries ( 39 ). Thus, as an adipose depot, the EAT exhibits specific upregulation in inflammatory processes and is susceptible to accentuation during CAD and other metabolic disturbances.

Shaihov-Teper and cols. ( 40 ) have demonstrated that proinflammatory, profibrotic, and proarrhythmic molecules are transported to the atria by the EAT. These molecules are associated with endothelial dysfunction and atherogenesis, supporting the hypothesis that EAT participates in the early stages of atheromatous plaque formation by changing the expression of uncoupling proteins.

Another possibility involving changes in expression of *UCP* genes is the occurrence of polymorphisms in the population; it is known that these polymorphisms – such as *UCP1 -3826 A/G, UCP 866 G/A,* and *UCP3 -55C/T* – may be associated with increased susceptibility to CAD. However, many studies have revealed that these polymorphisms have different effects depending on physical activity, lifestyle, and ethnicity ( 41 ). Finally, the CAD process is intricate and involves a complex interaction of genes, behavior, and environment, where the role of diet and nutritional deficiency states can interact. For example, vitamin A depletion is known to have an impact on metabolism and energy balance and, consequently, on male adiposity, exerting an evident influence on the genetic variants *SCARB1, UCP2* , and *UCP1* , although more studies are required for a complete understanding of these mechanisms ( 42 ).

Of note, most studies evaluating the association between the expression of UCP proteins and CAD have been carried out in rodent models. This highlights the importance of the present study, which evaluated the expression of these proteins in samples of adipose tissue in humans, specifically in patients of Mexican origin, who present a genetic load different than patients of Caucasian origin, in whom most human studies have been carried out.

### Limitations

A limitation of the present study was in its design, in which the patients considered as controls were not healthy subjects but, instead, patients with valve disease. This occurred due to difficulty in obtaining fatty tissue samples from healthy subjects. Although this study focused on UCP expression, other thermoregulatory molecules ( *e.g.* , PRDM16, PGC-1α, and PPARγ) may also be involved in the development of CAD and require further studies. Another potential limitation of the present study was the fact that the participants in the control group were younger than those in the CAD group, although we found no difference in age between the groups in correlation analysis.

In conclusion, the present study supports the hypothesis that a higher expression of UCPs in the EAT could act as a protective mechanism against the production of ROS and guard the myocardium against damage. This finding emphasizes the essential role that UCPs play in the adaptive phase of cardiac injury in the presence of metabolic disorders. The participation of UCPs could also be considered protective for the myocardium, although this effect should be analyzed over time in prospective studies.
